# Rotary tillage in rotation with plowing tillage improves soil properties and crop yield in a wheat-maize cropping system

**DOI:** 10.1371/journal.pone.0198193

**Published:** 2018-06-14

**Authors:** Li Zhang, Jing Wang, Guozhan Fu, Yonggan Zhao

**Affiliations:** 1 College of Agriculture, Henan University of Science and Technology, Luoyang, China; 2 Institute of Agricultural Resources and Regional Planning, Chinese Academy of Agricultural Sciences, Beijing, China; 3 Department of Energy and Power Engineering, Tsinghua University, Beijing, China; Fred Hutchinson Cancer Research Center, UNITED STATES

## Abstract

Soil rotational tillage is an effective measure to overcome the problems caused by long-term of a single tillage, but the effect of the interval time of rotational tillage practices is not very well understood. Therefore, we conducted a 3-year field study in a wheat-maize cropping system to evaluate the effects of rotary tillage (RT) in rotation with plowing tillage (PT) on soil properties in northern China. Four practices were designed as follows: 3 years of RT to a depth of 10–15 cm (3RT), 3 years of PT to a depth of 30–35 cm (3PT), 1 year of PT followed by 2 years of RT (PT+2RT), and 2 years of PT followed by 1 year of RT (2PT+RT). Within 20 cm of the surface soil, the 3RT treatment significantly increased the soil quality index (SQI) by 6.0%, 8.8% and 13.1%, respectively, relative to the PT+2RT, 2PT+RT and 3PT treatments. The improvement was closely related to the significant increase in the soil organic carbon (SOC) and available nutrients concentrations in the 0–20 cm depths and the improvement of soil invertase, urease, alkaline phosphatase and catalase activities in the topsoil (0–10 cm). However, the opposite effects were observed in the subsoil (20–40 cm). Compared with the 3RT treatment, the 3PT, 2PT+RT and PT+2RT treatments decreased soil bulk density, and significantly enhanced enzyme activities, resulting in an increase in SQI of 32.6%, 24.4% and 0.7%, respectively, especially in the 3PT and 2PT+RT treatments, the difference was significant. When averaged across to all soil depths, the SQI under the 3RT and 2PT+RT treatments was much higher than that under the other treatments. The yields of wheat and maize under the 2PT+RT treatment were 15.0% and 14.3% higher than those under the 3RT treatment, respectively. The 2PT+RT treatment was the most effective tillage practice. These results suggest that RT in rotation with PT could improve soil quality in the soil profile whilst enhancing crop yield after continuous RT, and the benefits were enhanced with an interval time of one year. Therefore, the 2PT+RT treatment could act as an effective method for both soil quality and crop yield improvement in a wheat-maize cropping system under straw incorporation conditions.

## Introduction

Long-term shallow tillage has formed a hard ploughing pan and increased subsoil compaction, which restrict root penetration and reduce water and nutrient uptake from deep layers, thus affecting drought resistance and yield improvement in most parts of northern China [1–3]. Deep tillage has been demonstrated to improve soil structure by breaking the dense soil layer, increasing the total porosity and creating a favourable environment for microbial metabolism and crop growth [4,5], but unfortunately it also markedly increases loss of soil organic carbon (SOC) and CO_2_ emission by destroying soil aggregate stability and increasing the rate of carbon mineralization [6,7].

Soil rotational tillage practice, a combination of at least two types of tillage practices from shallow tillage (e.g., no-tillage or rotary tillage (RT), to a depth <15 cm) and deep tillage (e.g., mouldboard plowing tillage (PT) or subsoiling to a depth >20 cm), has been considered as an effective method of resolving the shortcomings caused by continuous a single tillage [8,9]. Previous studies have shown that deep tillage (PT, subsoiling) in rotation with shallow tillage (no-tillage) significantly minimized soil compaction and favoured a better soil water status, which helped in crop development and increased water use efficiency [10,11]. However, there have been conflicting results regarding the appropriate interval time under different crop systems, soil type and climate [8,9,12–14]. The Huang-Huai-Hai Plain is one of the major grain-producing areas in China. RT after the summer maize (*Zea mays L*.) harvest and no-tillage after the winter wheat (*Triticum aestivum L*.) harvest have been widely adopted in this area due to savings time and cost. Years of continuous application of these tillage systems have resulted in subsoil compaction, limited root growth and reduced grain yield [1,3]. Thus, assessing the feasibility of RT in rotation with PT and determining the appropriate interval time are crucial for soil quality improvement and soil sustainable development.

However, based on our literature search, few studies have rarely focused on the effects of rotational tillage on soil properties in the Huang-Huai-Hai Plain and have primarily been limited to the effects on soil physico-chemical properties and crop yield [12–14], ignoring soil microbial characteristic. Soil enzymes participate in almost all biochemical reactions in soil and are sensitively response to the changes in tillage management in short time periods [15]. To our knowledge, the invertase, urease, alkaline phosphatase and catalase enzymes involved in the C, N and P cycling determined the nutrient quantity released for crop and soil microbial growth [16,17]. Therefore, these enzymes should be considered as valuable indicators for assessing the impacts of tillage practice on soil quality. We hypothesized that rotational tillage with RT and PT affect soil enzyme activity due to the change in the soil environment and the distribution of organic matter. The objective of this study was to evaluate the effects of RT in rotation with PT on the soil bulk density (BD), pH, SOC, available nutrients and enzyme activities in the 0–40 cm soil profile, assess soil quality through the soil quality index (SQI) and determine an appropriate interval time between RT and PT in the Huang-Huai-Hai Plain.

## Materials and methods

### Site description

The experiments were conducted from October 2009 to September 2012 in a field at the Henan University of Science and Technology (34°36′N, 112°24′E, 142 m above sea level) in Luoyang, Henan Province, China. This region has a semi-humid to semi-arid continental monsoon climate with a mean annual temperature of 14.1°C and a frost-free period of 204 days. The average annual precipitation is approximately 550 mm, of which approximately 60% falls during the summer maize growing period (June to September). The total annual precipitation in 2010, 2011 and 2012 were 485, 790 and 336 mm, respectively.

The experimental field was flat, and the soil was a yellow fluvo-aquic type with low fertility according to the FAO/UNESCO Soil Classification (FAO/UNESCO, 1993). The site was planted with winter wheat and summer maize within a double cropping system prior to the experiment. The tillage practices included RT after summer maize harvest and no-tillage after winter wheat harvest and were continuously performed for more than ten years. The initial physical and chemical properties of the 0–40 cm soil profile are presented in Table 1.

**Table 1:** Physical and chemical properties of the 0–40 cm soil profile at the experimental site.

Soil properties	Soil depth (cm)	
	0–20	20–40
Physical		
Sand (%)	37.4	31.7
Silt (%)	44.2	43.2
Clay (%)	18.4	25.1
Bulk density (g cm^-3^)	1.35	1.48
Maximum field capacity (%)	38.2	33.1
Chemical		
pH (H_2_O, 1:2.5)	7.86	8.01
Organic carbon (g kg^-1^)	6.3	3.1
Available N (mg kg^-1^)	75	44.8
Available P (mg kg^-1^)	9.2	4.5
Available K (mg kg^-1^)	120	109

### Experimental design and management

The experiment was set up in a randomized block design with three replicates. Each plot was 5 m wide and 30 m long. The experiment included four tillage treatments as follows: (i) 3 years of RT (3RT), (ii) 3 years of PT (3PT), (iii) 1 year of PT followed by 2 years of RT (PT+2RT), and (iv) 2 years of PT followed by 1 year of RT (2PT+RT). The sequence of different tillage practices is given in Table 2.

**Table 2:** Sequence of soil rotational tillage system from 2009 to 2012.

Tillage system	Year and crop					
	2009.10–2010.06 Winter wheat	2010.06–2010.10 Summer maize	2010.10–2011.06 Winter wheat	2011.06–2011.10 Summer maize	2011.10–2012.06 Winter wheat	2012.06–2012.10 Summer maize
3RT	NT	RT	NT	RT	NT	RT
3PT	NT	PT	NT	PT	NT	PT
PT+2RT	NT	PT	NT	RT	NT	RT
2PT+RT	NT	PT	NT	PT	NT	RT

RT and PT were implemented with base fertilizers and straw after summer maize harvest. NT was implemented with base fertilizers and straw after winter wheat harvest.

For the RT plots, the soil was rotated to a depth of 10–15 cm using a reverse-rotational rotary tiller (1GKN-200, Lianyungang Huayun Machinery Manufacturing Co., Ltd., Jiangsu, China). For the PT plots, the soil was ploughed to a depth of 30–35 cm using a tractor-mounted mouldboard plough (1LF7-450, Lovol Heavy Industry Co. Ltd., Shandong, China) with 40 cm between the ploughing strips. All the plots were harrowed to a depth of 10–15 cm by tractor (TT300, Mount Tai Tractor Factory Co. Ltd., Shandong, China) with an iron rake to ensure levelled ground for seedbed preparation.

Winter wheat was planted with a row spacing of 20 cm at a density of 270 seeds m^-1^ before 15 October each year. The wheat cultivar Zhongyu-10 was sown using a no-till planter (2BSF-5A, Zhengying Agricultural Machinery Co., Ltd., Shandong, China) with chisel-type openers and depth-controlling press wheels. Base fertilizer was applied at a rate of 105 kg ha^-1^ N (using urea, 46% N) plus DAP (diammonium phosphate, 18% N and 46% P_2_O_5_), 105 kg ha^-1^ P_2_O_5_ (using DAP) and 180 kg ha^-1^ K (using potassium sulphate, 50% K_2_O) based on the local fertilizer application practice. For all the plots, flood irrigation was applied to approximately 1350 m^3^ ha^-1^ one week before planting. At the wheat jointing stage, an additional 105 kg ha^-1^ urea was top-dressed and followed a second flood-irrigation with the same pre-planting water volume. Wheat was harvested using a standard combine harvester (4LZ-5, Kubota Agricultural Machinery (Suzhou) Co., Ltd., Jiangsu, China) in the early part of the following June, and the straw was then chopped and spread over the surface soil at the same time.

No-tillage was applied during the following summer maize production, during which a 5–8 cm wheat stubble height was left after the winter wheat was harvested.

Summer maize was direct-planted with a row spacing of 60 cm at a density of 75,000 plants ha^-1^ before 20 June each year. The maize cultivar Zhengdan-988 was sown using a precision planter (2BYF, Ninglian Machinery Manufacturing Co., Ltd., Shandong, China) along with fertilizer application. A compound fertilizer was then applied at 135 kg N ha^-1^, 90 kg P_2_O_5_ ha^-1^ and 180 kg K_2_O ha^-1^ using the same fertilizer as the one that was used for wheat production. For all the plots, flood irrigation was applied at approximately 1350 m^3^ ha^-1^ one day after planting. At the jointing and 12th-leaf stages of maize, an additional flood irrigation (1350 m^3^ ha^-1^) was applied. In addition, 135 kg ha^-1^ urea was top-dressed before the irrigation at the 12th-leaf stage. In late September, maize was harvested using a standard combine harvester (CB03, Lovol Heavy Industry Co. Ltd., Shandong, China), and the straw was then chopped and spread over the soil surface. Afterwards, the RT and/or PT practices were applied again with all residues using the same method as the one described for early October 2009.

For each crop production cycle, weeds, insects and diseases were controlled in a timely manner as required during the experimental period. Other management techniques were applied in a way that was consistent with the local agronomic practices.

### Soil sampling and measurement

Soil samples were collected during the third year of wheat-maize double cropping (March to October 2012). During the winter wheat season, the soils were sampled at the jointing (March 26), flowering (April 25) and maturity (June 1) stages. During the summer maize season, the soils were sampled at the jointing (July 5), flowering (August 5), filling (September 4) and maturity (October 7) stages. Soil samples were taken at a depth of 40 cm at 10-cm increments from five locations between the rows in each plot using a soil auger (3.8 cm diameter). Five samples from the same layer of the same treatment in each plot were mixed well into a composite sample.

The samples were passed through a 2-mm sieve and divided into two parts. To analyse the soil chemical properties, the samples were dried in a cool, dry and well-ventilated place, ground, and passed through a 1-mm sieve. The other samples were stored in the dark at 4°C for an analysis of the soil enzyme activity.

The soil BD was determined before the experiment in 2009 and after wheat and maize harvest in 2012 using the core ring method. Soil cores were taken up to a depth of 40 cm in layers of 10 cm from two locations in each plot. The soil pH was measured with a pH meter (FE-20K) at a soil:water ratio of 1:2.5 (w/v). The soil organic carbon (SOC) was determined by the K_2_CrO_4_–H_2_SO_4_ oxidation method. The soil available nitrogen (N) was estimated using the alkaline hydrolysis diffusion method. The soil available phosphorus (P) was extracted using 0.5 mol L^-1^ NaHCO_3_ and determined by the Mo-Sb colourimetric method. The soil available potassium (K) was extracted with 1 mol L^-1^ NH_4_OAc and analysed by the flame photometry method [18].

The invertase activity was analysed by incubating the soil samples with sucrose solution as a substrate at 37°C for 24 h, followed by measurements of the glucose contents using the 3,5-dinitrosalicylic acid colourimetric method. The invertase activity was reported in milligrams of C_6_H_12_O_6_ per gram of dry soil per day (mg C_6_H_12_O_6_ g^-1^ soil d^-1^) [19]. The urease activity was determined by incubating the soil sample with urea for 24 h at 37°C [20]. The amount of ammonium released from the soil was measured and expressed as mg NH_4_^+^-N g^-1^ soil d^-1^. The alkaline phosphatase activity was estimated by incubating the soil samples with p-nitrophenyl sodium phosphate solution (pH = 11) for 24 h at 37°C. The concentration of phenol that was released was measured colourimetrically at 420 nm, and the enzyme activity was expressed as mg p-nitrophenol released per gram of dry soil per day [21]. The catalase activity was determined using the KMnO_4_ volumetric method as described by Guan [22] and expressed as the change in H_2_O_2_ concentrations per gram of dry soil in 20 minutes. The enzyme activities were all expressed based on the air-dried weight of the soil.

### Calculations

The SQI was calculated using the following formula (Eq 1) [23]:

$$SQI=\sum_{i=1}^{n}W_{i}\times Q_{i}$$ (1)

where n is the number of variables; W_i_ is the weight for each indicator, which was calculated by its communality dividing by the sum of the communalities of all indicators [24]; and Q_i_ is the standardized data of all soil properties including soil BD, pH, nutrient contents and enzyme activities, which were normalized using the min-max method.

### Statistical analysis

All statistical tests were performed using SPSS software (v. 23.0, SPSS Inc., Chicago, IL). We used one-way ANOVA to test for effects of different tillage treatments on the BD, pH, SOC, available N, P and K contents within each depth. In addition, a statistical analysis involving crop growth stage and soil depth was conducted using a repeated measures ANOVA, where tillage practice, crop growth stage and soil depth were considered to be fixed effects; block was considered a random effect. A least significant difference test was used to assess the differences among the treatment means at the 0.05 level.

## Results

### Soil BD and pH response to different treatments

The soil BD was significantly affected by the rotational tillage practices (Table 3). In the 0–40 cm soil profile, the lowest BD was obtained in the 3PT treatment, followed by the 2PT+RT and PT+2RT treatments and there was no significant difference among the three treatments, except for at the 20–30 cm depth. However, the 3PT and 2PT+RT treatments had a negligible increase in soil pH at the 0–20 cm depths compared with the PT+2RT and 3RT treatments. and the opposite trend occurred at the deep depths (20–40 cm).

**Table 3:** Mean soil bulk density (BD), pH, soil organic carbon (SOC), and available N, P and K in the 0–40 cm soil depths under different treatments at the maturity stage of winter wheat and summer maize in 2012.

Soil depth (cm)	Treatment	BD (g cm^-3^)	pH	SOC (g kg^-1^)	Available N (mg kg^-1^)	Available P (mg kg^-1^)	Available K (mg kg^-1^)
0–10							
	3RT	1.20 a	7.69 a	7.71 a	76.71 b	18.88 a	265.0 a
	3PT	1.11 b	7.73 a	7.57 b	80.47 a	17.53 c	243.4 c
	PT+2RT	1.14 ab	7.70 a	7.67 ab	76.20 b	18.71 a	261.0 a
	2PT+RT	1.13 b	7.72 a	7.65 ab	79.87 a	18.07 b	249.0 b
10–20							
	3RT	1.36 a	7.76 a	6.51 a	65.63 a	7.24 a	207.1 a
	3PT	1.23 b	7.82 a	6.41 b	61.67 b	6.74 d	187.3 c
	PT+2RT	1.34 a	7.77 a	6.49 a	65.30 a	7.08 b	199.6 b
	2PT+RT	1.30 ab	7.78 a	6.42 b	62.21 b	6.98 c	195.6 b
20–30							
	3RT	1.46 a	8.00 a	4.67 c	47.57 c	3.65 d	120.8 c
	3PT	1.28 c	7.89 b	5.73 a	53.21 a	5.26 a	153.7 a
	PT+2RT	1.40 ab	7.96 ab	4.76 c	50.16 b	4.17 c	132.4 b
	2PT+RT	1.36 bc	7.91 b	5.29 b	51.90 a	5.04 b	149.5 a
30–40							
	3RT	1.48 a	8.04 a	3.52 b	42.33 c	1.68 c	95.6 c
	3PT	1.37 b	7.95 b	3.78 a	46.80 a	2.15 a	127.0 a
	PT+2RT	1.45 ab	8.03 a	3.57 b	44.48 b	1.73 c	112.2 b
	2PT+RT	1.39 b	7.99 ab	3.73 a	46.47 a	1.97 b	123.5 a
0–40				SOC stock (Mg ha^-1^)	Available N stock (kg ha^-1^)	Available P stock (kg ha^-1^)	Available K stock (kg ha^-1^)
	3RT	-	-	30.20 a	313.95 a	40.40 a	918.83 a
	3PT	-	-	28.87 b	297.55 c	37.51 c	872.97 b
	PT+2RT	-	-	29.37 ab	309.99 ab	39.32 ab	915.77 a
	2PT+RT	-	-	29.43 ab	306.98 b	39.15 b	912.39 a

Within each soil depth, the means followed by different lowercase letters are significantly different at the 0.05 level.

### Soil nutrient contents response to different treatments

The contents of all soil nutrients decreased with the increase in soil depth, and the magnitude of the decrease under the 3RT and PT+2RT treatments was greater than under the 3PT and 2PT+RT treatments (Table 3). At the 0–20 cm soil depths, the 3RT and PT+2RT treatments had significantly higher contents of SOC and available N, P and K than did the 3PT and 2PT+RT treatments, but the 3PT and 2PT+RT treatments had a significantly increased the available N content at the 10–20 cm depth compared to that in the 3RT and PT+2RT treatments. At the 20–40 cm depths, the soil nutrient contents among treatments exhibited a reverse trend. The 3PT treatment had a significantly higher soil nutrient contents than did the 3RT and PT+2RT treatments, but no significant difference in available N and K contents existed between the 3PT treatment and 2PT+RT treatments. When accumulated across the soil depths, the stocks of SOC and available N, P and K under the 3RT treatment significantly increased by 4.6%, 5.5%, 7.7% and 5.3%, respectively, compared with those under the 3PT treatment.

### Soil enzyme activities response to different treatments

**Fig 1:**
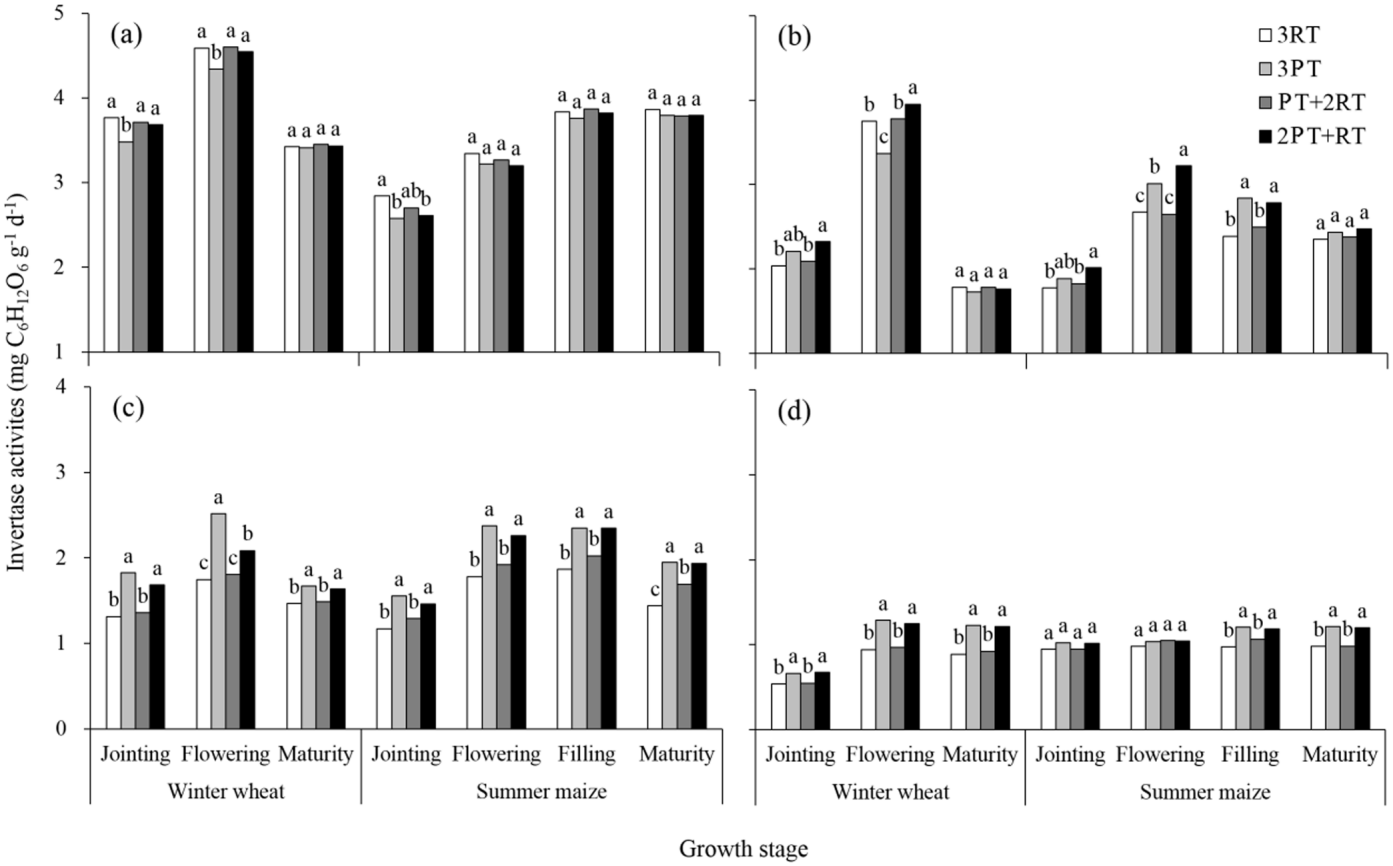
Invertase activity in the soil layers at 0–10 cm (a), 10–20 cm (b), 20–30 cm (c) and 30–40 cm (d) under different treatments during the winter wheat and summer maize seasons of 2012. Within each growth stage, the bars with different lowercase letters are significantly different at *P* < 0.05 according to the LSD test. 3RT, 3 years of RT to a depth of 10–15 cm; 3PT, 3 years of PT to a depth of 30–35 cm; PT+2RT, 1 year of PT followed by 2 years of RT; and 2PT+RT, 2 years of PT followed by 1 year of RT.

**Fig 2:**
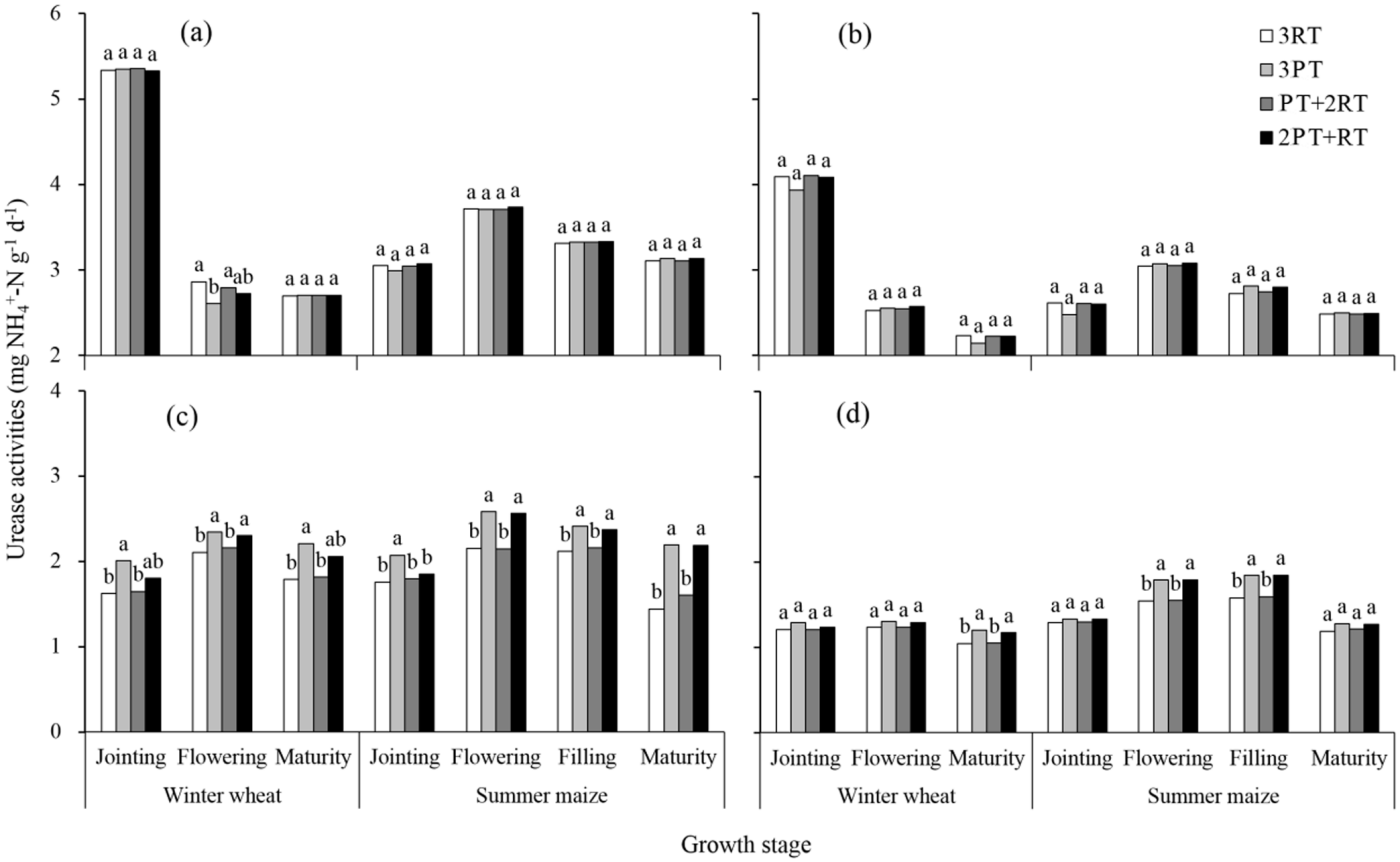
Urease activity in the soil layers from 0–10 cm (a), 10–20 cm (b), and 20–30 cm (c) and 30–40 cm (d) under different treatments during the winter wheat and summer maize seasons of 2012. Within one growth stage, bars with different lowercase letters are significantly different at *P* < 0.05 according to the LSD test. 3RT, 3 years of RT to a depth of 10–15 cm; 3PT, 3 years of PT to a depth of 30–35 cm; PT+2RT, 1 year of PT followed by 2 years of RT; and 2PT+RT, 2 years of PT followed by 1 year of RT.

**Fig 3:**
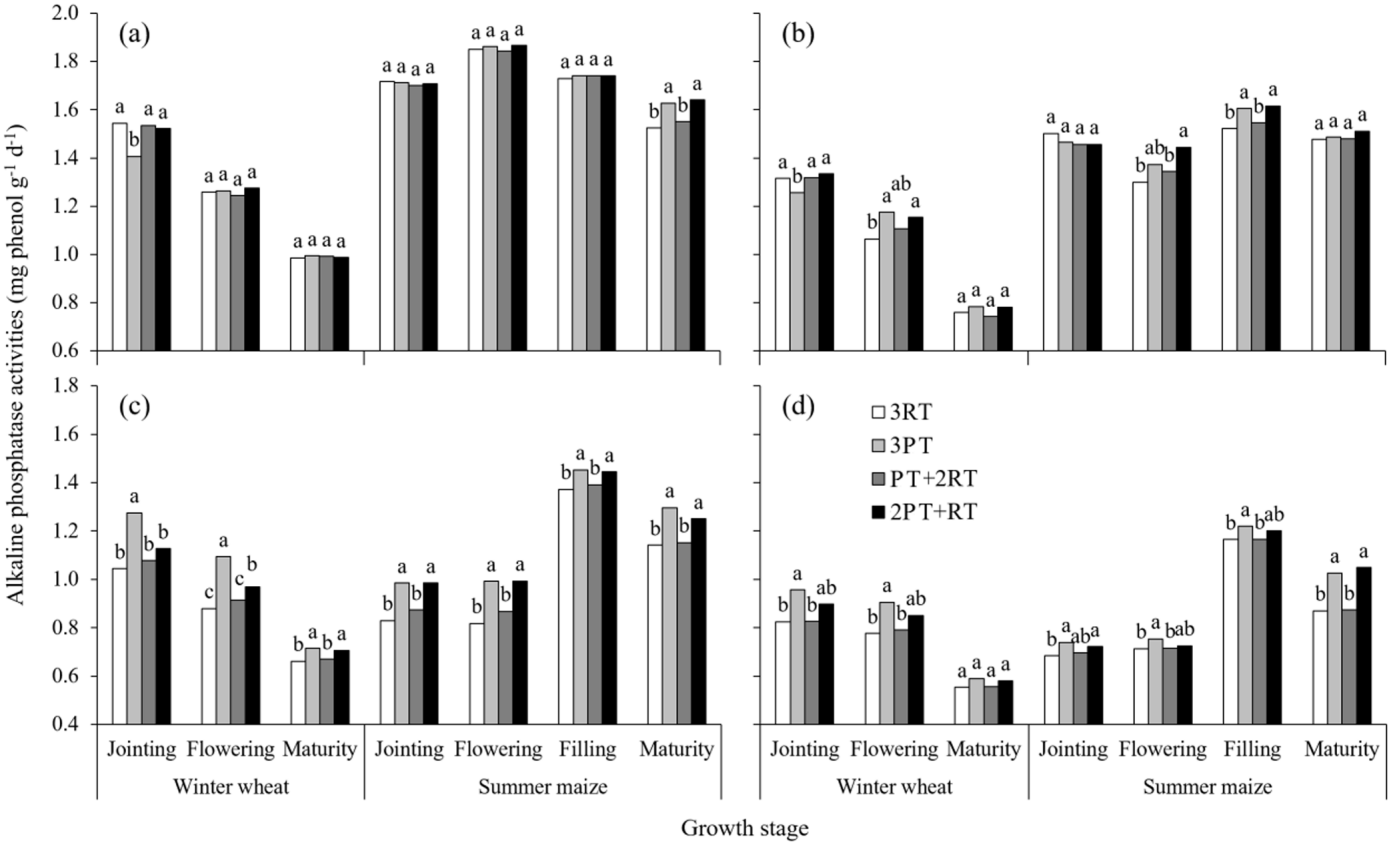
Alkaline phosphatase activity in soil layers from 0–10 cm (a), 10–20 cm (b), and 20–30 cm (c) and 30–40 cm (d) under different treatments during the winter wheat and summer maize seasons of 2012. Within one growth stage, bars with different lowercase letters are significantly different at *P* < 0.05 according to the LSD test. 3RT, 3 years of RT to a depth of 10–15 cm; 3PT, 3 years of PT to a depth of 30–35 cm; PT+2RT, 1 year of PT followed by 2 years of RT; and 2PT+RT, 2 years of PT followed by 1 year of RT.

**Fig 4:**
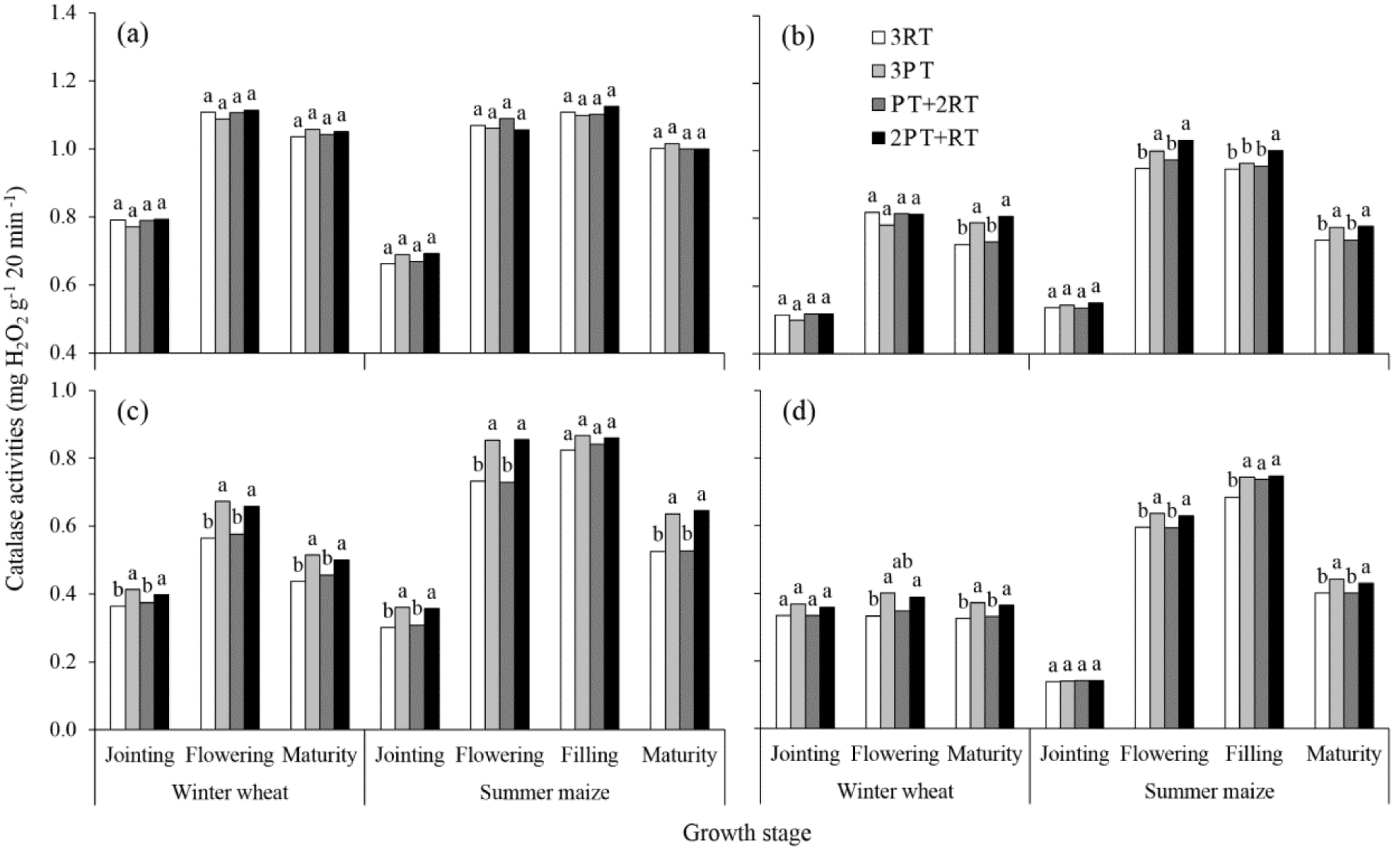
Catalase activity in the soil layers at 0–10 cm (a), 10–20 cm (b), and 20–30 cm (c) and 30–40 cm (d) under different treatments during the winter wheat and summer maize seasons of 2012. Within each growth stage, bars with different lowercase letters are significantly different at *P* < 0.05 based on the LSD test. 3RT, 3 years of RT to a depth of 10–15 cm; 3PT, 3 years of PT to a depth of 30–35 cm; PT+2RT, 1 year of PT followed by 2 years of RT; and 2PT+RT, 2 years of PT followed by 1 year of RT.

The tillage practices, growth period and soil depth and their interactions significantly affected the soil enzyme activities at all soil depths (Table 4). Over the course of the growth stages, the soil enzyme activities generally decreased with the increasing soil depths (Figs 1–4). In the summer maize growing period, the enzyme activities generally increased at the early stage, peaked, and then decreased. The changing trend of the invertase and catalase activities during the winter wheat growing period was similar with that of summer maize growing season. However, the highest values for the urease and alkaline phosphatase activities were found at the jointing stage, and these levels decreased throughout the wheat growth period. Significant differences in the invertase activities were found among treatments at the 10–30 cm depths (Fig 1b and 1c). Compared to the 3RT treatment, the 3PT and 2PT+RT treatments increased the invertase activity at the 20–30 cm depth by 31.8% and 24.7% over the growth stages, respectively. At the 30–40 cm depth, significant differences in the invertase activity among the 3RT, 3PT and 2PT+RT treatments were found only during the winter wheat season. However, there was a decrease in the invertase activity at some wheat stages from the 0–20 cm depths (Fig 1a and 1b).

**Table 4:** Repeated measures ANOVA *P* value for the effects of treatment (T), crop growth stage (G), soil depth (D), and their interactions on soil invertase activity, urease activity, alkaline phosphatase activity and catalase activity.

Variable	df	Inverse activity	Urease activity	Alkaline phosphatase activity	Catalase activity
T	3	<0.001	<0.001	<0.001	<0.001
G	6	<0.001	<0.001	<0.001	<0.001
D	3	<0.001	<0.001	<0.001	<0.001
T×G	18	<0.001	<0.001	<0.001	<0.001
T×D	9	<0.001	<0.001	<0.001	<0.001
G×D	18	<0.001	<0.001	<0.001	<0.001
T×G×D	54	<0.001	<0.001	<0.001	<0.001

The differences in urease activities among tillage practices differed significantly at the 20–30 cm depth (Fig 2c), and at some stages of summer maize at the 30–40 cm depths (Fig 2d). Compared to the 3RT treatment, the 3PT and 2PT+RT treatments increased the urease activity at the 20–30 cm depths by 21.8% and 16.6%, respectively. However, the opposite trend occurred at some growth stages at the 0–20 cm depth (Fig 2a and 2b), but the differences among treatments were not significant.

The differences in alkaline phosphatase activities among tillage practices were found primarily at the 20–30 cm depth and at some stages at the 30–40 cm depth (Fig 3c and 3d). Compared with that of the 3RT treatment, the average alkaline phosphatase activity at the 20–40 cm depths in the 3PT and 2PT+RT treatments increased by 13.5% and 9.4%, respectively, and the 3PT treatment had a significantly higher value than did the 2PT+RT treatment at the 20–30 cm depth. At the 0–20 cm depths, the value under the 3PT and 2PT+RT treatments was significantly higher than that under the 3RT treatment throughout the summer maize season (Fig 3a and 3b). However, there was a significant decrease in the alkaline phosphatase activity in the 3PT treatment at the jointing stage of winter wheat at the 0–20 cm depths relative to the other three treatments.

Additionally, significant differences were found in the catalase activities among tillage practices at the 20–30 cm depths and at some stages at the 10–20 and 30–40 cm depths (Fig 4b–4d). Compared to the 3RT treatment, the catalase activity at the 20–30 cm depths under the 3PT and 2PT+RT treatments increased by 15.2% and 14.1%, respectively. However, there was an increase in the catalase activity of the 3RT treatment at the jointing and flowering stages of winter wheat at the 0–20 cm depths, relative to the 3PT and 2PT+RT treatments (Fig 4a and 4b).

### Soil quality index response to different treatments

Tillage practices significantly affected SQI value in the soil profile (Table 5). At the 0–10 cm and 10–20 cm depths, the SQI under the 3RT treatment was significantly higher than under the 2PT+RT and 3PT treatments. However, at the 20–30 cm and 30–40 cm depths, the 2PT+RT and 3PT treatments had a much greater SQI than did the 3RT and PT+2RT treatments, and there was no significant difference between the 3PT and 2PT+RT treatments. When averaged across all soil depths, the SQI value among treatments varied in the order 3RT>2PT+RT>3PT>PT+2RT. The value under the 3RT and 2PT+RT treatments distinctly increased by 4.5% and 3.2%, relative to the PT+2RT treatment.

**Table 5:** Mean of soil quality index (SQI) at the 0–40 cm soil depths under different tillage treatments at the maturity of winter wheat and summer maize in 2012.

Treatment	0–10 cm	10–20 cm	20–30 cm	30–40 cm	0–40 cm^a)^
3RT	0.880 a	0.603 a	0.273 c	0.122 b	0.469 a
3PT	0.797 d	0.514 c	0.371 a	0.152 a	0.458 bc
PT+2RT	0.842 b	0.557 b	0.276 c	0.121 b	0.449 c
2PT+RT	0.814 c	0.550 b	0.339 b	0.152 a	0.464 ab

Within each soil depth, the means followed by different lowercase letters are significantly different at the 0.05 level.

^a)^ The value of SQI was the means in the 0–40 cm depths.

### Crop grain yield response to different treatments

Grain yields of wheat and maize under the 3PT and 2PT+RT treatments were significantly higher than those under the 3RT and PT+2RT treatments, but the yields were not significantly different between the 3PT and 2PT+RT treatments (Table 6). On averaged for the two seasons, the yield under the 3PT and 2PT+RT treatments increased by 15.5% and 14.6% compared to that under the 3RT treatment, and by 9.0% and 8.2% compared to that under the PT+2RT treatment.

**Table 6:** Changes in grain yield of winter wheat and summer maize under different treatments in 2012.

Treatment	Wheat yield (kg ha^-1^)	Maize yield (kg ha^-1^)	Mean (kg ha^-1^)
3RT	7083.09 c	8329.13 c	7706.11 c
3PT	8170.51 a	9624.92 a	8897.72 a
PT+2RT	7582.65 b	8743.03 b	8162.84 b
2PT+RT	8147.02 a	9522.47 a	8834.74 a

Within each soil depth, the means followed by different lowercase letters are significantly different at the 0.05 level.

## Discussion

In our study, at the 0–20 cm depths, the 3RT treatment produced a clear increase in the concentration of SOC and available N, P and K, relative to deep tillage practices (the 3PT, 2PT+RT and PT+2RT treatments). However, further improvement in the soil nutrient concentrations at the 20–40 cm depth was observed in the 3PT, 2PT+RT and PT+2RT treatments. The difference might be ascribed to the distribution of straw and base fertilizers in the soil profile. Previous studies [3, 25] have also shown that the vertical gradient of soil properties decreased under deep tillage used due to the increase of the soil nutrient concentrations in the 20–60 cm soil layer by burying most organic material in the deep layers. These results suggest that the residues act as the main sources of subsoil nutrients and their distribution in the soil profile largely determines the degree of soil fertility at different depths. Furthermore, soil BD was much higher in the 20–40 cm layers (Table 1) than that in the topsoil, where there was poor soil aeration, helping to reduce rate of organic matter mineralization and SOC accumulation [26,27]. In addition, the increase in soil nutrient concentrations might be related to the amount of residues added to the soil. Previous studies have revealed that changes in soil structure and nutrient distribution affect root growth and spatial distribution [28,29]. In our study, deep PT drastically decreased BD and created a less-limited soil penetration resistance for root growth and higher biomass production. When the storage of soil nutrients in the soil profile were averaged, however, the 3PT treatment had the significantly lowest value compared to that of the other treatments, possibly because deep tillage accelerated C and N mineralization by destroying the structure of soil macro-aggregates and enhancing soil metabolic intensity with improvement in aeration in the soil profile [7,10]. Compared with the 3PT treatment, the stocks of soil nutrients under the 2PT+RT and PT+2RT treatments were significantly higher. A previous study carried in northwest China also found that rotational tillage could increase the stocks of organic matter and total N both in both surface and subsurface soil, suggesting that shallow tillage in rotation with deep tillage (subsoiling) is the best practice for the continuous cropping of wheat fields [30]. However, the PT+2RT treatment had a weaker ability to reduce subsoil compaction and could not maintain soil nutrient homogeneous distribution, which suggests that PT should be applied every other year in rotation with shallow tillage in the winter wheat-summer maize system in northern China. González-Prieto et al. [31] studied the effect of different tillage practices on soil properties in a calcic haploxeralf in a leguminous-cereal rotation system and found that the extent and duration of effects on soil properties under PT treatment could be maintained for 2 years; otherwise, the cone index and BD sharply increased. However, Pierce et al. [8] analysed data from long-term field experiments on a Capac loam in a corn-rye system and found that the improvement of most soil physical quality parameters and the stratification ratio of soil nutrients under rotational tillage practice returned to initial levels after approximately 4 or 5 years of PT. These conflicting results have been attributed to differences in cropping systems, type of shallow tillage and climatic conditions. In our study, the soil rotational tillage system included RT and PT, rather than no tillage and PT, and the cropping system was a wheat-maize rotation system rather than a corn-green manure system. This indicated that the soil was tightly squeezed by mechanical compaction, such as by RT, wheat planters and harvesters during the winter wheat season. In addition, the structure of soil aggregates was broken down by the blades of the rotary tiller [3], which might result in micro-aggregates filling into porosity, thereby accelerating soil compaction under precipitation and irrigation [32] and shortening the duration of the improvement effects on soil properties under the deep plowing tillage.

Soil enzymes participate in almost all biochemical reactions in soil and are sensitive response to changes in tillage management in the short term [15–17]. The enzyme activity presented obvious seasonal variations (Figs 1–4). The average activities of the invertase and catalase enzymes at the 0–40 cm depths gradually increased with crop growth and peaked at the wheat-flowering stage and maize-filling stage. This trend was consistent with the findings of Zhang et al. [33], who reported a higher enzyme activity during the vigorous growth period because of improved increases in root biomass and root exudates, which provided more carbon source for micro-organisms growth and reproduction. However, the urease activity in the 0–20 cm layer gradually decreased with wheat growth, which was not consistent with the findings of Jin et al. [34]. This decrease possibly was explained by N fertilizer being applied at the early stage as a result of urea hydrolysis [35]. In addition, the seasonal changes in the activity were more obvious in the 0–20 cm layer than in the 20–40 cm layer, especially during the wheat growing season. These differences might due to the distinct change in environmental factors, such as soil temperature and humidity in the topsoil (0–20 cm) during the wheat growing season.

In addition to the growth period and soil depth, enzyme activities were significantly affected by the tillage practices (Table 4). In the winter wheat growing season. the activities of all enzymes in the 0–10 cm layer under the 3RT treatment were generally the higher than those under deep tillage practices These results are similar to those of other authors [36,37], who reported that urease and phosphatase activities in the topsoil significantly increased under shallow tillage, primarily due to the highest soil nutrient contents available for enzyme metabolism. However, the average enzyme activity in the 0–40 cm layer over the two crop seasons was the highest under the 2PT+RT treatment, followed by the 3PT treatment and lowest under the 3RT treatment. This result is likely due to the differences in soil physical and chemical properties. The changes in the quantities of soil nutrients greatly influenced the microbial process [38,39]. The 2PT+RT treatment had much greater stocks of soil organic matter and available nutrients in the soil profile than did the 3PT treatment, which might satisfy the needs for soil microorganism’s growth and reproduction. Soil BD was significantly negatively correlated with soil enzyme activity [40]. Soil BD in the soil profile was much lower under the 2PT+RT treatment than under the 3RT treatment. The improvement of soil physical properties created a more favourable microbial microhabitat to release extracellular enzymes and increase their activities. Other studies have shown that the activity of soil hydrolase was closely associated with root biomass due to the partial origin of soil enzymes from root exudates [41]. Thus, the decrease of soil densification might have a slightly positive effect on the improved soil enzyme activity through the increased root density [3].

Soil quality is the result of the integration of soil physical, chemical and biological properties. Comprehensive evaluation the impact of different treatments on soil quality is imperative to make correct decision for cultivated soil development [42]. SQI is an effective method for comprehensively evaluating and quantitatively assessing the impact of agricultural management on soil quality [43]. Our results showed that the 3RT and PT+2RT treatments significantly increased the SQI value in the upper 20 cm (Table 5), but had an apparent opposite effect on SQI at the 20–40 cm depth compared with the 3PT and PT+2RT treatments. Similar to the studies of Yang and Wander [44], who reported that the shallow tillage only improved soil fertility in the first few centimetres of the surface, relative to deep tillage, there was no significant difference in the soil profile. In our study, the average value of SQI in the soil profile under the 3RT and 2PT+RT treatments was higher than under the PT+2RT and 3PT treatments. This suggest that 3RT and 2PT+RT treatments could maintain soil quality, but considering the yields of wheat and maize, the 2PT+RT treatment might be a win-win measure maintaining soil sustainable development and ensuring food security in the Huang-Huai-Hai Plain.

## Conclusions

Our results showed that the 3RT treatment increased soil compaction and improved soil nutrients concentrations and enzyme activities in the 0–10 cm layer, but significantly decreased these factors in the 20–40 cm layer relative to the results of the 3PT treatment. The combinations of PT and RT, especially 2PT+RT, not only decreased the soil bulk density, increased the homogenization of soil nutrient spatial distribution but also improved the soil quality at a depth of 40 cm and increased grain yield. Thus, in a wheat-maize cropping system, deep plowing tillage should be used after one year of RT application to improve the soil quality. Clearly, further study is needed to test the effects of the interval time of soil rotational practices on soil quality and grain yield in long-term field experiments and different cropping systems.

## Supporting information

S1 Data Data in Table 1. (XLSX)

S2 Data Data in Table 3. (XLSX)

S3 Data Data in Table 4. (XLSX)

S4 Data Data in Table 5. (XLSX)

S5 Data Data in Table 6. (XLSX)

S6 Data Data in Fig 1. (XLSX)

S7 Data Data in Fig 2. (XLSX)

S8 Data Data in Fig 3. (XLSX)

S9 Data Data in Fig 4. (XLSX)

## References

[1] Yang XM, Drury CF, Reynolds WD, Tan CS. Impacts of long-term and recently imposed tillage practices on the vertical distribution of soil organic carbon. Soil Tillage Res. 2008;100: 120–124. doi: 10.1016/j.still.2008.05.003

[2] Bengough AG, McKenzie BM, Hallett PD, Valentine TA. Root elongation, water stress, and mechanical impedance: a review of limiting stresses and beneficial root tip traits. J Exp Bot. 2011;62: 59–68. doi: 10.1093/jxb/erq350 21118824 10.1093/jxb/erq350

[3] Mu X, Zhao Y, Liu K, Ji B, Guo H, Xue Z, et al Responses of soil properties, root growth and crop yield to tillage and crop residue management in a wheat–maize cropping system on the North China Plain. Eur J Agron. 2016;78: 32–43. doi: 10.1016/j.eja.2016.04.010

[4] Varsa EC, Chong SK, Abolaji JO, Farquhar DA, Olsen FJ. Effect of deep tillage on soil physical characteristics and corn (*Zea mays L*.) root growth and production. Soil Tillage Res. 1997;43: 219–228. doi: 10.1016/S0167-1987(97)00041-X

[5] Sun X, Ding Z, Wang X, Hou H, Zhou B, Yue Y, et al Subsoiling practices change root distribution and increase post-anthesis dry matter accumulation and yield in summer maize. PLOS ONE. 2017;12: e0174952 doi: 10.1371/journal.pone.0174952 28384233 10.1371/journal.pone.0174952 PMC5383055

[6] Cheng-Fang L, Dan-Na Z, Zhi-Kui K, Zhi-Sheng Z, Jin-Ping W, Ming-Li C, et al Effects of tillage and nitrogen fertilizers on CH_4_ and CO_2_ emissions and soil organic carbon in paddy fields of central China. PLOS ONE. 2012;7: e34642 doi: 10.1371/journal.pone.0034642 22574109 10.1371/journal.pone.0034642 PMC3344821

[7] Devine S, Markewitz D, Hendrix P, Coleman D. Soil aggregates and associated organic matter under conventional tillage, no-tillage, and forest succession after three decades. PLOS ONE. 2014;9: e84988 doi: 10.1371/journal.pone.0084988 24465460 10.1371/journal.pone.0084988 PMC3896348

[8] Pierce FJ, Fortin M-C, Staton MJ. Periodic plowing effects on soil properties in a no-till farming system. Soil Sci Soc Am J. 1994;58: 1782–1787. doi: 10.2136/sssaj1994.03615995005800060029x

[9] López-Fando C, Pardo MT. Changes in soil chemical characteristics with different tillage practices in a semi-arid environment. Soil Tillage Res. 2009;104: 278–284. doi: 10.1016/j.still.2009.03.005

[10] Rashidi M, Keshavarzpour F. Effect of different tillage methods on grain yield and yield components of maize (*Zea mays L*.). Int J Agric Biol. 2007;2: 274–277.

[11] Hou X, Jia Z, Han Q, Li R, Wang W, Li Y. Effects of rotational tillage practices on soil water characteristics and crop yields in semi-arid areas of north-west China. Soil Res. 2011;49: 625–632. doi: 10.1071/SR11143

[12] Jin H, Hongwen L, Xiaoyan W, Mchugh A, Wenying L, Huanwen G, et al The adoption of annual subsoiling as conservation tillage in dryland maize and wheat cultivation in northern China. Soil Tillage Res. 2007;94: 493–502. doi: 10.1016/j.still.2006.10.005

[13] Carter MR, Sanderson JB, Ivany JA, White RP. Influence of rotation and tillage on forage maize productivity, weed species, and soil quality of a fine sandy loam in the cool–humid climate of Atlantic Canada. Soil Tillage Res. 2002;67: 85–98. doi: 10.1016/S0167-1987(02)00043-0

[14] Hou X, Li R, Jia Z, Han Q, Wang W, Yang B. Effects of rotational tillage practices on soil properties, winter wheat yields and water-use efficiency in semi-arid areas of north-west China. Field Crops Res. 2012;129: 7–13. doi: 10.1016/j.fcr.2011.12.021

[15] Paz-Ferreiro J, Trasar-Cepeda C, Leiros MC, Seoane S, Gil-Sotres F. Biochemical properties in managed grassland soils in a temperate humid zone: modifications of soil quality as a consequence of intensive grassland use. Biol Fert Soils. 2009;45: 711–722.

[16] Burns RG. Enzyme activity in soil: location and a possible role in microbial ecology. Soil Biol Biochem. 1982;14: 423–427. doi: 10.1016/0038-0717(82)90099-2

[17] Caldwell BA. Enzyme activities as a component of soil biodiversity: a review. Pedobiologia. 2005;49: 637–644. doi: 10.1016/j.pedobi.2005.06.003

[18] Lu RK. Analytical methods of soil agricultural chemistry. Beijing, China: China Agricultural Science and Technology Press; 2000.

[19] Gu Y, Wang P, Kong CH. Urease, invertase, dehydrogenase and polyphenoloxidase activities in paddy soil influenced by allelopathic rice variety. Eur J Soil Biol. 2009;45: 436–441. doi: 10.1016/j.ejsobi.2009.06.003

[20] Kandeler E, Gerber H. Short-term assay of soil urease activity using colorimetric determination of ammonium. Biol Fert Soils. 1988;6: 68–72. doi: 10.1007/BF00257924

[21] Ge GF, Li ZJ, Zhang J, Wang LG, Xu MG, Zhang JB, et al Geographical and climatic differences in long-term effect of organic and inorganic amendments on soil enzymatic activities and respiration in field experimental stations of China. Ecol Complexity. 2009;6: 421–431. doi: 10.1016/j.ecocom.2009.02.001

[22] Guan SY. Soil Enzymes and its methodology. Beijing, China: Agricultural Press; 1986.

[23] Liu Z, Rong Q, Zhou W, Liang G. Effects of inorganic and organic amendment on soil chemical properties, enzyme activities, microbial community and soil quality in yellow clayey soil. PLOS ONE. 2017;12: e0172767 doi: 10.1371/journal.pone.0172767 28263999 10.1371/journal.pone.0172767 PMC5338777

[24] Shukla MK, Lal R, Ebinger M. Determining soil quality indicators by factor analysis. Soil Tillage Res. 2006;87: 194–204. doi: 10.1016/j.still.2005.03.011

[25] Linh TB, Sleutel S, Vo Thi G, Le Van K, Cornelis WM. Deeper tillage and root growth in annual rice-upland cropping systems result in improved rice yield and economic profit relative to rice monoculture. Soil Tillage Res. 2015;154: 44–52. doi: 10.1016/j.still.2015.06.011

[26] Smith KA, Ball T, Conen F, Dobbie KE, Massheder J, Rey A. Exchange of greenhouse gases between soil and atmosphere: interactions of soil physical factors and biological processes. Eur J Soil Sci. 2003;54: 779–791. doi: 10.1046/j.1351-0754.2003.0567.x

[27] Poll C, Marhan S, Ingwersen J, Kandeler E. Dynamics of litter carbon turnover and microbial abundance in a rye detritusphere. Soil Bio Biochem. 2008;40: 1306–1321. doi: 10.1016/j.soilbio.2007.04.002

[28] Zhai L, Xu P, Li S, Xie R, Zhai L, et al Effects of deep vertical rotary tillage on dry matter accumulation and grain yield of summer maize in the Huang-Huai-Hai Plain of China. Soil Tillage Res. 2017;170: 167–174. doi: 10.1016/j.still.2017.03.013

[29] Li H, Gao H, Wu H, Li W, Wang X, He J. Effects of 15 years of conservation tillage on soil structure and productivity of wheat cultivation in northern China. Aust J Soil Res. 2007;45(5): 344–350. doi: 10.1071/SR07003

[30] Wang Y, Li J, Bai W. Effects of rotational tillage systems on soil production performance in wheat-maize rotation field in Loess Platform region of China. Transactions of the CSAE. 2015; 31(1): 107–116.

[31] González-Prieto S, Díaz-Raviña M, Martín A, López-Fando C. Effects of agricultural management on chemical and biochemical properties of a semiarid soil from central Spain. Soil Tillage Res. 2013;134: 49–55. doi: 10.1016/j.still.2013.07.007

[32] Six J, Elliott ET, Paustian K. Soil macroaggregate turnover and microaggregate formation: a mechanism for C sequestration under no-tillage agriculture. Soil Bio Biochem, 2000; 32(14): 2099–2103. https://doi.org/10.1016/S0038-0717(00)00179-6.

[33] Zhang X, Ma L, Gilliam FS, Wang Q, Li C. Effects of raised-bed planting for enhanced summer maize yield on rhizosphere soil microbial functional groups and enzyme activity in Henan Province, China. Field Crops Res. 2012;130(2): 28–37. doi: 10.1016/j.fcr.2012.02.008

[34] Jin K, Sleutel S, Buchan D, De Neve SD, Cai DX, Gabriels D, et al Changes of soil enzyme activities under different tillage practices in the Chinese Loess Plateau. Soil Tillage Res. 2009;104: 115–120. doi: 10.1016/j.still.2009.02.004

[35] Tabatabai MA, Bremner JM. Assay of urease activity in soils. Soil Bio Biochem. 1972;4: 479–487. doi: 10.1016/0038-0717(72)90064-8

[36] Hamido SA, Kpomblekou-A K. Cover crop and tillage effects on soil enzyme activities following tomato. Soil Tillage Res. 2009;105: 269–274. doi: 10.1016/j.still.2009.09.007

[37] Seddaiu G, Iocola I, Farina R, Orsini R, Iezzi G, Roggero PP. Long term effects of tillage practices and N fertilization in rainfed Mediterranean cropping systems: durum wheat, sunflower and maize grain yield. Eur J Agron. 2016;77: 166–178. doi: 10.1016/j.eja.2016.02.008

[38] Bandick AK, Dick RP. Field management effects on soil enzyme activities. Soil Biol Biochem. 1999;31: 1471–1479. doi: 10.1016/S0038-0717(99)00051-6

[39] Roldán A, Salinas-García JR, Alguacil MM, Caravaca F. Changes in soil enzyme activity, fertility, aggregation and C sequestration mediated by conservation tillage practices and water regime in a maize field. Appl Soil Ecol. 2005;30: 11–20. doi: 10.1016/j.apsoil.2005.01.004

[40] Deng SP, Tabatabai MA. Effect of tillage and residue management on enzyme activities in soils. Biol Fert Soils. 1996;22: 202–207. doi: 10.1007/BF00382513

[41] Geng Y, Dighton J, Gray D. The effects of thinning and soil disturbance on enzyme activities under pitch pine soil in New Jersey Pinelands. Appl Soil Ecol. 2012;62: 1–7. doi: 10.1016/j.apsoil.2012.07.001

[42] Karlen DL, Andrews SS, Doran JW. Soil quality: current concepts and applications. Adv Agron. 2001;74: 1–40. doi: 10.1016/S0065-2113(01)74029-1

[43] Qi Y, Darilek JL, Huang B, Zhao Y, Sun W, Gu Z. Evaluating soil quality indices in an agricultural region of Jiangsu Province, China. Geoderma. 2009;149: 325–334. doi: 10.1016/j.geoderma.2008.12.015

[44] Yang XM, Wander MM. Tillage effects on soil organic carbon distribution and storage in a silt loam soil in Illinois. Soil Tillage Res. 1999;52: 1–9. doi: 10.1016/S0167-1987(99)00051-3

